# Evaluation of Pathogenetic and Immunological Properties of a Vietnamese Isolate of Porcine Reproductive and Respiratory Syndrome Virus of Vietnam in Experimentally Infected Piglets

**DOI:** 10.3390/vetsci12111084

**Published:** 2025-11-14

**Authors:** Hiep Van Dang, Anh Duc Truong, Nhu Thi Chu, Hoai Thi Phan, Hieu Minh Nguyen, Quoc Khanh Dam, Linh Phuong Nguyen, Kien Van Le, Hao Thi Vu, Le Thi Hai Vo, Tuong Dinh Nguyen, Ha Thi Thanh Tran, Hoang Vu Dang

**Affiliations:** 1 Department of Biochemistry and Immunology, National Institute of Veterinary Research, 86 Truong Chinh, Kim Lien, Hanoi 100000, Vietnam; hiepcctythanhhoa@gmail.com (H.V.D.); truonganhduc84@gmail.com (A.D.T.); chunhuk58tyc@gmail.com (N.T.C.); phanhoai271@gmail.com (H.T.P.); nmhieu.vp@gmail.com (H.M.N.); damkhanh699@gmail.com (Q.K.D.); ngphuonglinhwhale2811@gmail.com (L.P.N.);; 2 Sub-Department of Livestock and Animal Health, Thanh Hoa Department of Agriculture and Rural Development, 63 Le Van Huu, Tan Son, Thanh Hoa 440000, Vietnam; lekien.k10@gmail.com; 3 Faculty of Agriculture, Forestry and Fisheries, Nghe An University, 51 Ly Tu Trong, Ha Huy Tap, Vinh 460000, Vietnam; vietnamlevth@nau.edu.vn (L.T.H.V.); tuongnd@nau.edu.vn (T.D.N.)

**Keywords:** PRRS, cytokine, clinical sign, immune response

## Abstract

This study aimed to evaluate the pathogenic potential of the 2024 Vietnam PRRSV genotype 2 (NA01/2024, isolate). Fifteen 6-week-old piglets were intramuscularly inoculated with 1 × 10^5.5^ TCID_50_/mL of PRRSV, while fifteen control piglets remained uninfected. Blood and nasal swabs were collected every 3 days until 21 days post-infection (dpi). Necropsies were performed on piglets at 6, 15, and 21 dpi. Infected piglets exhibited fever, blue ear, weight loss, respiratory distress, diarrhea, and leucopenia between 3 and 12 dpi. PRRSV was detected in serum and nasal secretions up to 21 dpi, peaking between 6 and 9 dpi. Seroconversion began at 6 dpi, with the highest antibody titers at 21 dpi. Virus load was highest in lung tissues at all intervals, while the spleen and lymph nodes showed higher viral load at later stages. Routine blood tests indicated a slight decrease in red blood cells, hemoglobin, and reticulocytes, along with a notable increase in monocytes in infected piglets. Cytokine levels (IFN-γ, IL-6, and IL-10) in serum and lung tissues were significantly higher in infected piglets compared to controls. Additionally, the PRRSV infection triggered innate immune responses, including cytokines, growth factors, and chemokines in whole blood and tissues such as IFN-α, IFN-β, TNF-α, IL-1β, etc. These findings highlight the pathogenicity of the Vietnam PRRSV NA01/2024 isolate and its impact on the immune response, providing insights into PRRSV infection mechanisms and strategies for prevention and control.

## Simple Summary

Porcine reproductive and respiratory syndrome virus (PRRSV) causes huge economic losses in the global swine industry. Little is known about the effects of newly HP-PRRS outbreak in Vietnam on the pathogenicity, disease severity, inflammatory mediators, and antibody responses. Fifteen 6-week-old piglets were intramuscularly inoculated with 1 × 10^5.5^ TCID_50_/mL of the newly HP-PRRS genotype 2 isolate (NA01/2024), while 15 remained uninfected as controls. Infected piglets showed fever, blue ear, respiratory distress, diarrhea, and weight loss between 3 and 12 days post-infection (dpi). Viral RNA was detected in serum and nasal swabs up to 21 dpi, with the highest levels at 6–9 dpi. The lungs exhibited the greatest viral load, followed by the spleen and lymph nodes. Seroconversion occurred from 6 dpi, reaching peak antibody titers at 21 dpi. Hematological analysis showed mild anemia, leucopenia, and increased monocytes. Cytokine profiling revealed elevated IFN-γ, IL-6, IL-10, IFN-α, IFN-β, TNF-α, and IL-1β in infected piglets. These findings confirm the pathogenic potential of the NA01/2024 strain and its ability to trigger strong immune and inflammatory responses in pigs.

## 1. Introduction

Porcine reproductive and respiratory syndrome (PRRS) is a significant pig disease caused by a small RNA virus belonging to the order Nidovirales and the family Arteriviridae [1,2]. Currently, PRRS virus (PRRSV) is divided into two species, including the *Betaarterivirus suid* 1 (formerly PRRSV-1 or genotype 1, Type 1 or European—EU) and *Betaarterivirus suid* 2 (formerly PRRSV-2, or genotype 2, Type 2 or North American—NA) types, which roughly share 60% nucleotide identity [3]. PRRS is recognized by the World Organisation for Animal Health as notifiable disease with a significant socioeconomic impact [4,5]. PRRS poses a significant threat to pig health, causing both reproductive and respiratory challenges. Reproductive effects include abortions, stillborn or mummified piglets and elevated mortality rates among neonatal piglets. Across all age groups, clinical signs may manifest as fever, sneezing, coughing, pneumonia, pyrexia, and anorexia. The severity of symptoms can be influenced by age, immune status, and the presence of concurrent infections [4,5]. The PRRS was first discovered in the United States in 1987 and subsequently reported in Europe during the 1990s [6]. Since then, it has spread globally, becoming a significant concern for the swine industry worldwide [6]. The PRRS virus was first reported in the early 1990s in Asia [2]. In 2007, a new strain known as “highly pathogenic” (HP) PRRS emerged in China and Vietnam, and the HP-PRRS virus strain is characterized by its high morbidity and mortality rates and subsequently spread to other Asian countries such as Korea, Thailand, etc., leading to significant economic losses in the region’s swine industry [1,7,8].

In Vietnam, it has been reported that the PRRSV may have entered through imported pigs in the late 1990s from the USA [7,8]. HP-PRRSV was officially noted in Vietnam in Hai Duong province in 2007 and then spread out across the whole country [7]. Based on whole-genome sequencing analysis, the HP-PRRSV prevailing in Vietnam has a partial deletion in nsp2 gene compared to the North American genotype 2 [7]. The genomic analysis of the PRRSV from the northern part of Vietnam revealed 99% similarity to the PRRSV strains from China [7]. Research on the seroprevalence of PRRSV in the Mekong Delta region (MDR) revealed that 9.3% (37 out of 397) of pigs tested were positive for the virus [7]. Based on ORF5 sequencing analysis, type 2 PRRS (NA-PRRS) is divided into nine different lineages distributed across various geographical regions. In China, 4 lineages are currently circulating, including lineage 1 (sub-lineage 1.8, NADC30-like strains and sub-lineage 1.5, NADC34-like strains), lineage 3 (QYYZ-like strains), lineage 5 (VR2332-like strains), and lineage 8 (sub-lineage 8.1, Chinese classical PRRSV strains and sub-lineage 8.7, highly pathogenic the PRRS strains (HP-PRRSV)) [9,10,11,12]. In Vietnam, several studies on the molecular epidemiology of PRRS virus before 2021 indicated that three lineages are currently circulating in Vietnam, including lineage 8 (sub-lineage 8.7, highly pathogenic PRRS strains—HP-PRRSV), lineage 5.1, and lineage 1 (NADC-like group, sub-lineage 1.4), in which sub-lineage 8.7 and lineage 5.1 have been circulating since 2007, and sub-lineage 1.4 was newly discovered in 2021 [7,13,14].

Although HP-PRRS has recently re-emerged in Vietnam, limited information is available regarding its impact on viral pathogenicity, disease progression, host immune responses, and antibody dynamics. This study investigated the newly emerged HP-PRRSV strain NA01/2024 (lineage 8.7, sub-lineage SX2009/2009-like) isolated in Vietnam. Experimental infection in piglets was conducted to evaluate its effects on clinical manifestations, viral load and shedding, hematological changes, cytokine expression, and specific antibody responses. The findings provide insights into the pathogenicity and immune response dynamics associated with the recent HP-PRRSV outbreak in Vietnam

## 2. Materials and Methods

### 2.1. Ethical Statement

This study complied with the institutional rules for the care and use of laboratory animals and a protocol approved by the Ministry of Agriculture and Rural Development (MARD) Vietnam (TCVN 8400-21:2014) [15].

### 2.2. Virus and Virus-Neutralization (VN) Test

The PRRSV strain was isolated from the lung of a pig that died in the north-central region of Vietnam in 2024. The NSP2 sequence of NA01/2024 exhibited the characteristic deletion pattern commonly observed in highly pathogenic PRRSV (HP-PRRSV) strains from Asia. Phylogenetic analysis based on the ORF5, NSP2, and full-genome sequences placed NA01/2024 within lineage 8.7, sub-lineage SX2009/2009-like, and closely related to recent HP-PRRSV field isolates from northern Vietnam and southern China (GenBank Acc. No. PX475808 for ORF5 gene and PX475809 for NSP2 gene). The HP-PRRSV NA01/2024 strain was isolated and propagated in MARC-145 cells and maintained in Dulbecco’s Modified Eagle’s Medium (DMEM; Invitrogen, Waltham, MA, USA) supplemented with 10% fetal bovine serum (FBS; Thermo Scientific, Waltham, MA, USA) and antibiotics (100 U/mL penicillin, 10 mg/mL streptomycin, and 25 µg/mL amphotericin B; Sigma-Aldrich, St. Louis, MO, USA) at 37 °C in a humidified atmosphere containing 5% CO_2_. The HP-PRRSV strain NA01/2024 was used for piglet inoculation after three passages on the MARC-145 cell line. The stock virus titer for experimental infection was 5 × 10^6.5^ TCID_50_/mL, and the inoculum was prepared at a concentration of 1 × 10^5.5^ TCID_50_/mL for infection.

A virus-neutralization (VN) test against HP-PRRSV isolate NA01/2024 strain was done on Marc-145 cells as previously described [16]. The VN antibody titter was defined as the reciprocal of the highest serum dilution that completely inhibited cytopathic effects in all wells. Samples with titers greater than 2.0 (log_2_) were considered positive [17].

### 2.3. Animal Experiment

The piglets used in this study were obtained from a PRRSV-free farm located in Hanoi, Vietnam. Prior to the infection experiment, thirty healthy, weaned, 6-week-old Large White × Dutch Landrace crossbred piglets were confirmed to be free of PRRSV by enzyme-linked immunosorbent assay (ELISA; IDEXX Laboratories Inc., Westbrook, ME, USA). Real-time RT-PCR was also performed to verify the absence of PRRSV nucleic acids in nasal swabs and blood samples (the RT-PCR method is described in Section 2.5). Additionally, RT-PCR and serological tests were conducted to confirm that all piglets were negative for classical swine fever virus (CSFV), African swine fever virus (ASFV), pseudorabies virus (PRV), swine influenza virus (SIV), Mycoplasma hyopneumoniae, and porcine parvovirus (PPV).

The 6-week-old specific pathogen-free (SPF) piglets were randomly divided into two groups of fifteen animals each. Piglets in the infected group were intramuscularly inoculated with 1 mL of the HP-PRRSV NA01/2024 strain at a titer of 10^5.5^ TCID_50_/mL per piglet, while the control group received 1 mL of phosphate-buffered saline (PBS). All piglets were monitored daily for mental state, rectal temperature, anorexia, severe depression, sneezing, coughing, tachypnea, labored or abdominal breathing, and other symptoms such as diarrhea, conjunctivitis, ear reddening, skin cyanosis, lameness, and mortality. Clinical signs were scored according to the method described by Li et al. [18].

Rectal temperatures were recorded daily before feeding throughout the experimental period. Nasal swabs and blood samples were collected at 0, 3, 6, 9, 12-, 15-, 18-, and 21 days post-infection (dpi) to evaluate viral genome loads, serum antibody titers, and cytokine mRNA expression levels. Three pigs from each group were randomly selected and euthanized at 6 and 15 dpi following HP-PRRSV challenge. Blood, heart, liver, spleen, lung, kidney, and pooled lymph node samples (including intestinal, inguinal, submaxillary, bronchial, gastrohepatic, and mediastinal lymph nodes) were collected for further analyses. Tissue samples were stored at −80 °C for RNA extraction and quantitative PCR analysis. The remaining pigs in each group were euthanized at 21 dpi. Serum antibody responses were quantified using a commercial PRRSV enzyme-linked immunosorbent assay (ELISA) kit (IDEXX Laboratories, Atlanta, GA, USA) according to the manufacturer’s instructions.

### 2.4. Routine Blood Tests

Blood samples were collected from the jugular vein, using HTM K2 EDTA tubes, every 3 days before feeding in the morning. Blood samples were placed into HTM K2 EDTA (Ethylenediamine tetra Acid) tubes (HTM, Vietnam) containing 2 mL anticoagulant solution (EDTA). The automatic biochemical analyzer for veterinary diagnostics (DMT-120VP, Chengdu Seamaly Technology Co., Ltd., Chengdu, Sichuan, China) was used to perform complete blood cell count analysis [red blood cells (RBCs), white blood cells (WBCs), hemoglobin levels, lymphocytes, and platelet counts]. All procedures for the analysis of blood count were performed at the Pet One Clinic in Hanoi, Vietnam. 

### 2.5. Detection of Pathogen Load by qRT-PCR

Viral RNA from the samples was extracted using a Viral RNA Mini Kit (QIAGEN Straße 1, Hilden 4072, Germany) according to the manufacturer’s instructions. The primer and probe sequences used for HP-PRRSV detection in this experiment are listed in Table 1 and were applied at a working concentration of 10 μM. The quantity of HP-PRRSV RNA in piglet samples was determined by quantitative real-time RT-PCR (qRT-PCR) following WOAH and TCVN (Vietnam National Standards) guidelines [5,18], using the SuperScript™ III Platinum^®^ One-Step Quantitative RT-PCR System (Invitrogen, Carlsbad, CA, USA). Reactions were performed on a QuantStudio 5 Real-Time PCR System (Thermo Scientific, Waltham, MA, USA) with the following thermal profile: reverse transcription at 50 °C for 30 min; initial activation at 95 °C for 15 min; followed by 40 cycles of denaturation at 95 °C for 10 s and annealing at 60 °C for 50 s. All samples were tested in duplicate. The viral RNA copy number was calculated using the standard curve method. Samples with Ct values < 35 were considered positive.

### 2.6. RNA Extraction, cDNA Synthesis, and qPCR

Total RNA was extracted from whole blood and tissue samples using TRIzol reagent (Invitrogen, Carlsbad, CA, USA) according to the manufacturer’s protocol. Up to 2 µg of total RNA was treated with 1 U of DNase I and 1 µL of 10× reaction buffer (Thermo Fisher Scientific, Waltham, MA, USA) at 37 °C for 30 min to remove genomic DNA contamination. The reaction was terminated by adding 1 µL of 50 mM EDTA and heating at 65 °C for 10 min. Complementary DNA (cDNA) was synthesized using the Maxima First Strand cDNA Synthesis Kit (Thermo Fisher Scientific) following the manufacturer’s instructions. Quantitative real-time PCR (qRT-PCR) was performed to determine the expression levels of cytokine and chemokine genes using gene-specific primers (Table 2) and 2× AmpiGene SYBR Green Master Mix (Enzo Life Sciences, Farmingdale, NY, USA) on a QuantStudio 5 Real-Time PCR System (Thermo Fisher Scientific). β-actin was used as the internal reference gene, and relative expression levels were calculated using the 2^−ΔΔCt^ method after normalization to β-actin expression [19].

### 2.7. Cytokine Assays

Lung tissues collected during necropsy were analyzed for cytokine concentrations as previously described [20]. Briefly, 1 g of lung tissue was homogenized in 1 mL of phosphate-buffered saline (PBS; 1:1, *w*/*v*) using an X620 tissue homogenizer (CAT, M. Zipperer GmbH, Ballrechten-Dottingen, Germany). The homogenates were centrifuged at 3000× *g* for 10 min, and the supernatants were stored at −80 °C until cytokine analysis. Serum samples were collected at 0, 3, 6, 9, 12-, 15-, 18-, and 21-day post-infection (dpi). The concentrations of cytokines (IL-6, IL-10, and IFN-γ) in both lung homogenates and serum were quantified using commercial ELISA kits (Invitrogen; IL-6, Cat. No. ESIL6; IL-10, Cat. No. KSC0101; IFN-γ, Cat. No. ES7RS) according to the manufacturer’s instructions. All assays were performed in triplicate.

### 2.8. Statistical Analysis

All experimental data were analyzed using IBM SPSS Statistics software (version 25.0; IBM Corp., Chicago, IL, USA). Results are presented as the mean ± standard error of the mean (SEM) from at least three independent replicates. Statistical differences among groups were assessed using Duncan’s multiple comparison test, and *p* values less than 0.05 were considered statistically significant.

## 3. Results

### 3.1. Clinical Evaluation

**Figure 1:**
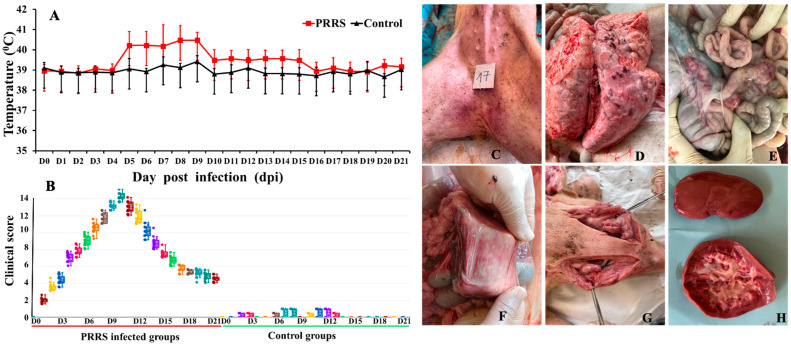
Clinical score outcome in piglets. (**A**) Rectal temperature (mean ± SD) and (**B**) clinical score (mean ± SD) of piglets infected with PRRSV NA01/2024 virus strain and control groups; colors just separate of day infection. (**C**–**H**) Gross lesions in pigs that died of infection with the PRRSV. The lesions of the skin hemorrhage, lung, mesenteric lymph nodes in the small intestine, stomach, lymph nodes, and kidney of pigs inoculated with 10^5.5^ TCID50/mL of the virus are shown here and the pictures were taken by camera with 18 MP resolution, starting at ISO80 and with F1.78 at the long range (**C**–**H**).

Throughout the entire study period, the control piglets showed no clinical signs associated with PRRSV infection. In contrast, piglets infected with the PRRSV NA01/2024 strain exhibited evident clinical symptoms, including labored breathing, transient anorexia, and occasional episodes of diarrhea. The rectal temperatures of PRRSV-infected pigs increased significantly at 5 days post-infection (dpi). The body temperature of 8 out of 15 infected pigs exceeded 41 °C between 5 and 9 dpi and remained above 40.5 °C until 10 dpi; temperatures then began to decline to around 40.2 °C between 10 and 12 dpi. From 15 to 21 dpi, the rectal temperature of PRRSV-infected pigs did not show a significant increase (Figure 1A).

Clinical scores in piglets were determined by monitoring observable clinical signs, including mental state, rectal temperature, anorexia, severe depression, sneezing, coughing, tachypnea, labored or abdominal breathing, and other symptoms such as mortality, diarrhea, conjunctivitis, reddening of the ears, skin cyanosis, and lameness. These clinical signs in piglets infected with the HP-PRRSV NA01/2024 strain were mostly observed between 4 and 15 dpi (Figure 1B), and no piglets died during the study. The average clinical score in infected animals exceeded eight from 7 dpi onward.

### 3.2. Gross Lesion

All surviving pigs were necropsied at 21 dpi for evaluation of gross lesions. Gross examination of PRRSV-infected pigs revealed reddish-purple discoloration of the skin on the ears, legs, and abdomen, accompanied by skin blisters on the body (Figure 1C). Pulmonary hemorrhages were observed as patchy and mottled areas, with the lungs appearing collapsed against the rib cage. A thick, mucus-like exudate was present along the lung edges, and purulent material was visible on the cut surface of the lungs. Pleural adhesions were frequently noted in affected pigs (Figure 1D). Hemorrhages were also observed in various segments of the intestines of diseased pigs, with the most severe lesions located in the mesenteric lymph nodes. The hemorrhage or congestion gave the intestinal lymph nodes a marbled appearance. Gastric congestion, mucosal hemorrhages, and suffusions were also evident in the stomach (Figure 1E,F). The superficial inguinal lymph nodes showed swelling and edema compared with normal controls, with petechial hemorrhages on the surface. Upon incision, mottled hemorrhagic areas resembling marble veining were observed on the cut surface of the lymph nodes, along with signs of degeneration (Figure 1G). Pinpoint hemorrhages, resembling pinpricks, were present on the kidneys, and upon bisection, severe hemorrhage was observed in the renal pelvis (Figure 1H). Pericardial adhesions were present, and the thoracic cavity contained a substantial amount of turbid white fluid. The heart appeared flattened, and the myocardium was flaccid. Due to lung damage and the pig’s sustained high oxygen demand, the heart was overworked, resulting in the lesions described above. Alterations in other organs varied depending on the stage of disease progression and the presence of secondary infections.

### 3.3. Viral RNA Load and Antibody

**Figure 2:**
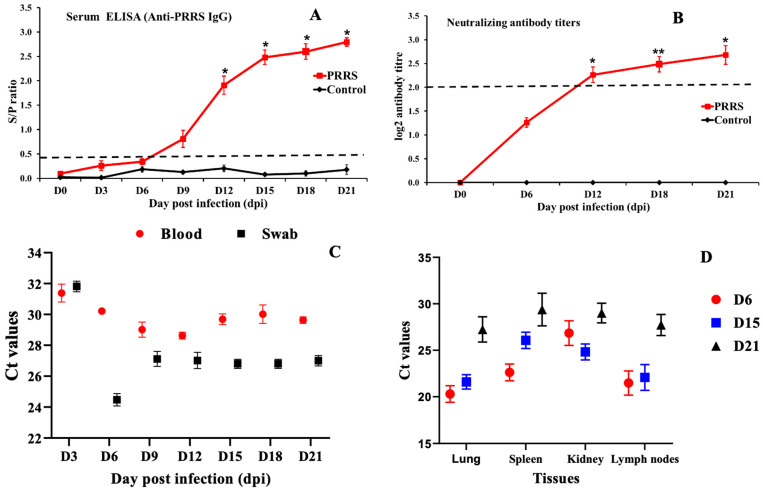
Virus load and Antibody responses against PRRSV infections. (**A**) ELISA measured the PRRSV-specific antibody titer in pigs. The dashed line indicates the cutoff value with S/P ratio of 0.4. (**B**) VN antibodies titer (log2) specific to the PRRSV NA01/2024 strain by day and group; (**C**) Viral load in blood and swab; and (**D**) viral loads in four tissues after PRRSV NA01/2024 challenges. Viral RNA of PRRSV in samples were evaluated by qPCR. Error bars represent standard deviations. Data were analyzed by one-way analysis of variance (ANOVA), and mean comparisons were performed using Duncan’s multiple range test. Significant differences between treatment and control groups were indicated as *: *p* < 0.05, **: *p* < 0.01.

The PRRSV-specific antibody levels in the serum of piglets infected and non-infected with the PRRSV NA01/2024 strain are shown in Figure 2A. At 0, 3, 6, 9, 12, 15, 18, and 21 days post-infection (dpi), antibody levels in serum samples were determined by ELISA. All pigs tested negative for PRRSV-specific antibodies upon arrival and at the time of inoculation, with S/P ratios below 0.4 in the ELISA assay. In the PRRSV-infected group, 40% of the animals were seropositive at 6 dpi, and by 9 dpi, all pigs had developed detectable antibody responses. The differences in the kinetics and magnitude of S/P ratios between the infected and control groups were statistically significant (*p* < 0.05) (Figure 2A). Virus-neutralizing antibodies (Figure 2B) were first detected at 6 dpi in pigs inoculated with the PRRSV NA01/2024 strain, and their levels peaked at 21 dpi. No antibodies were detected in the control group throughout the experimental period.

The viral load of PRRSV in whole blood and nasal swabs of pigs is presented in Figure 2B. All animals tested negative for PRRSV before inoculation (0 dpi). At 3 dpi, viral loads detected in the blood and nasal swabs of infected piglets increased significantly, reaching their peak at 6 dpi compared with the control group. Viral RNA levels in nasal swabs remained higher throughout the entire experimental period in PRRSV-infected pigs. The observed differences in viral RNA dynamics between nasal swabs and whole blood over time were statistically significant (*p* < 0.1). In contrast, viral RNA was not detected in any samples from the control group during the study period. In addition, the viral load levels in various organs of PRRSV-infected pigs, including the spleen, lungs, kidneys, and lymph nodes, showed noticeable variation. At 6 dpi, viral loads in these tissues of piglets infected with the PRRSV NA01/2024 strain were higher than those at 15 and 21 dpi, with the lungs exhibiting the highest viral titers compared with the spleen, kidneys, and lymph nodes. However, these differences among organs were not statistically significant (Figure 2C). Furthermore, PRRSV genomic RNA was detected in the heart, liver, intestine, stomach, and brain of infected piglets, providing strong evidence of the widespread tissue tropism of the pathogenic PRRSV NA01/2024 strain. The virus was successfully re-isolated and propagated from nasal swab, whole blood, and lung samples of infected pigs at 6 dpi in Marc-145 cells to confirm the infection.

### 3.4. Blood Count Analysis

In piglets infected with the PRRSV NA01/2024 strain, red blood cell (RBC) counts and hemoglobin levels were markedly decreased from 3 to 18 days post-infection (dpi) compared with the control group (Table 1). These values remained low but showed a gradual increase by 18 dpi. Pigs inoculated with PRRSV exhibited a transient, mild reduction in RBC and hematocrit levels at 3 and 12 dpi; however, these values remained within the normal physiological range. The lowest hemoglobin levels were recorded between 9 and 12 dpi. In contrast, hemoglobin levels in pigs infected with HP-PRRSV remained comparable to those of the control group throughout the experiment (Table 1). Hematocrit values in HP-PRRSV-infected pigs were also similar to those observed in the control group. White blood cell (WBC) counts in HP-PRRSV-infected pigs decreased significantly from 3 to 9 dpi and then increased from 12 dpi until the end of the experiment (Table 3). Monocyte, eosinophil, and neutrophil counts were significantly higher in infected pigs than in control pigs, although they remained within the normal range (Table 3). In contrast, lymphocyte numbers in infected pigs showed a slight decrease from 3 to 9 dpi, followed by a modest increase at 12 dpi compared with the control group (Table 3).

### 3.5. Cytokine mRNA Expression Profiles in Whole-Blood

**Figure 3:**
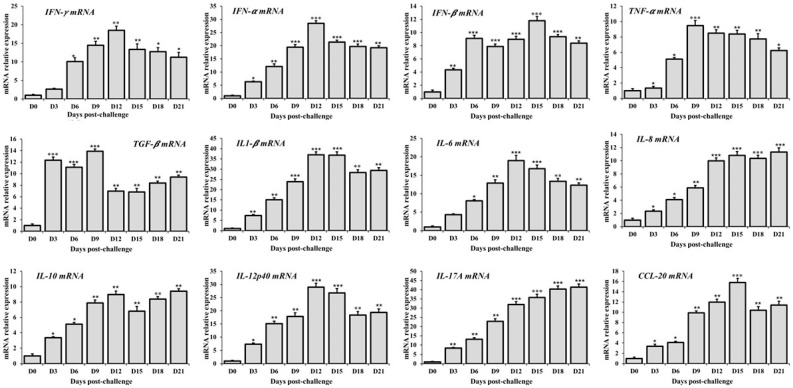
Cytokine mRNA expression in whole blood of piglets infected with the HP-PRRSV. Quantitative RT-PCR analyzed the expression levels of cytokine mRNA in whole blood. The relative levels of cytokine mRNA expression are expressed as the mean fold-increase in the virus-infected groups compared to those of the control group; error bars represent standard deviations. Data were analyzed by one-way analysis of variance (ANOVA), and mean comparisons were performed using Duncan’s multiple range test. Significant differences between treatment and control groups were indicated as *: *p* < 0.05, **: *p* < 0.01 and ***: *p* < 0.001.

In this study, we analyzed the expression of pro-inflammatory cytokines, including IFN-γ, TNF-α, IL-1β, IL-6, and IL-12p40, in the whole blood of pigs infected with the HP-PRRSV strain NA01/2024. As shown in Figure 3, the expression levels of IFN-γ, IFN-α, IL-1β, IL-6, and IL-12p40 mRNA were significantly increased, reaching their highest levels at 12 days post-infection (dpi), with fold changes of 18.49-, 28.39-, 36.98-, and 28.98-fold, respectively, before gradually declining thereafter. The expression of IL-8 and IL-17A mRNA was significantly upregulated from 3 dpi and peaked at 21 dpi, showing 11.32-fold and 41.40-fold increases, respectively (Figure 3). The pro-inflammatory cytokine TNF-α and the growth factor TGF-β gradually increased in HP-PRRSV-infected pigs from 3 to 9 dpi and then declined by 21 dpi, although their expression levels remained higher than those of the control group. The anti-inflammatory cytokine IL-10 was significantly upregulated following infection, reaching its peak at 12 dpi, after which its expression gradually decreased but remained elevated through 21 dpi. The expression of the innate immune factor IFN-β was also significantly upregulated, with the highest expression observed at 15 dpi (11.82-fold change; Figure 3). Similarly, the chemokine CCL20 mRNA was markedly upregulated after HP-PRRSV infection, peaking at 15 dpi with a 15.82-fold increase (Figure 3). Overall, these results indicate that a broad range of cytokines and chemokines involved in innate immunity were strongly induced in HP-PRRSV-infected pigs, suggesting the activation of an effective innate immune response following infection.

### 3.6. Cytokine mRNA Expression Profiles in Tissues

**Figure 4:**
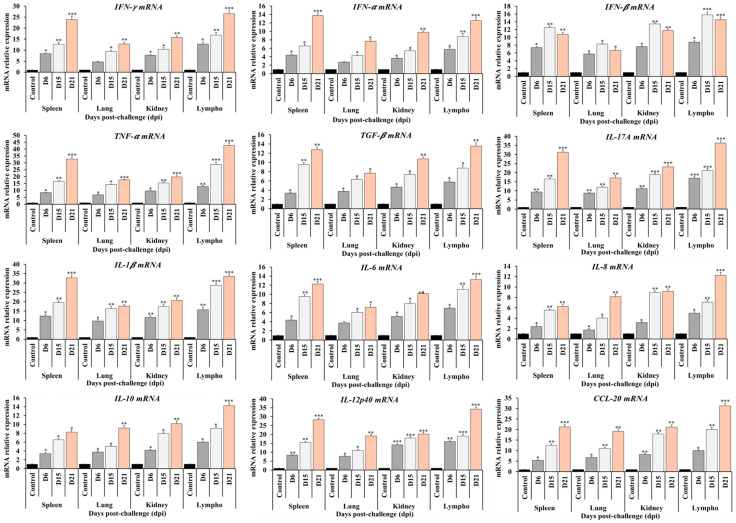
Cytokine mRNA expression in the spleen, lung, kidney and lymph nodes of piglet infected with HP-PRRSV NA01/2024 strain isolated from outbreak of Vietnam in 2024-at 6 dpi, 15 dpi, and 21 dpi. Quantitative RT-PCR analyzed the expression levels of cytokine mRNA in tissues. The relative levels of cytokine mRNA expression are expressed as the mean fold-increase in infected groups to control group; error bars represent standard deviations. Data were analyzed by one-way analysis of variance (ANOVA), and mean comparisons were performed using Duncan’s multiple range test. Significant differences between treatment and control groups were indicated as *: *p* < 0.05, **: *p* < 0.01 and ***: *p* < 0.001.

At 6, 15, and 21 days post-infection (dpi), samples of the lung, kidney, spleen, and lymph nodes were collected. The expression levels of innate immunity markers and cytokines, including IFN-α, IFN-β, IFN-γ, TNF-α, TGF-β, IL-1β, IL-6, IL-8, IL-10, IL-12p40, IL-17A, and CCL20 mRNA, were evaluated by RT-qPCR in tissues from pigs infected with the HP-PRRSV NA01/2024 isolate. As shown in Figure 4, the expression levels of these innate immune markers and cytokines in the tissues of infected pigs were higher than those in the control group and reached their highest levels at 21 dpi, except for IFN-β, which peaked at 15 dpi. Among the analyzed tissues, the lymph nodes exhibited the highest expression levels of innate immunity markers and cytokines compared to the lung, kidney, and spleen (Figure 4).

### 3.7. Cytokine Assay Analysis

**Figure 5:**
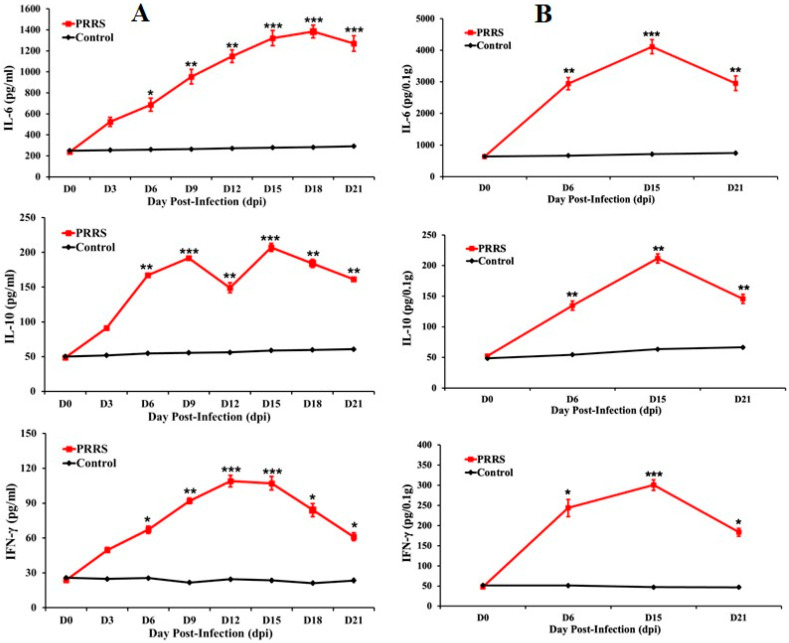
Proinflammatory cytokine levels in lungs and sera. The lung tissue samples were collected at 0-, 6-, 15- and 21-day post-HP-PRRSV NA01/2024 challenge, and the serum samples were collected from the pigs at 0, 3, 6, 9, 12, 15, 18 and 21 dpi in each group. (**A**) Levels of IL-6, IL-10, and IFN-γ in homogenates of lung were evaluated by commercial ELISA kit. (**B**) ELISA detected levels of IL-6, IL-10, and IFN-γ in serum samples. Data were analyzed by one-way analysis of variance (ANOVA), and mean comparisons were performed using Duncan’s multiple range test. Significant differences between treatment and control groups were indicated as *: *p* < 0.05, **: *p* < 0.01 and ***: *p* < 0.001.

At 0, 3, 6, 9, 12, 15, 18, and 21 days post-infection (dpi), blood samples were collected, and lung samples were obtained at 0, 6, 15, and 21 dpi. The expression levels of IL-10, IL-6, and IFN-γ in serum were determined by ELISA, as shown in Figure 5. The results indicated that the cytokine profiles following HP-PRRSV infection exhibited similar trends. The levels of IL-6, IL-10, and IFN-γ increased after infection, peaked at various time points, and then declined by 21 dpi. The concentration of IL-6 in serum reached its highest level at 18 dpi (1300 pg/mL; *p* < 0.001), while IFN-γ peaked at 12 dpi (100 pg/mL; *p* < 0.001) and IL-10 at 15 dpi (200 pg/mL; *p* < 0.001). Similar expression patterns of IL-6, IL-10, and IFN-γ were observed in both lung and serum samples (Figure 5). The tested cytokines were significantly elevated in all infected animals compared with the controls. This expression pattern was consistent with the distribution of gross lung lesions, as strong correlations were observed between lung pathology and the concentrations of IL-6, IL-10, and IFN-γ in piglets infected with the PRRSV NA01/2024 strain.

## 4. Discussion

PRRSV is an emerging viral infectious disease. PRRS virus (PRRSV) can affect all age groups [21]. Reproductive impairment or failure is more evident in sows or gilts but can affect boars [20]. PRRSV was first reported in Vietnam in late 2007 and then has been continuing as a major disease in pigs’ population of Vietnam and swine industry in the world, it is causing to huge economic losses [7]. Currently, PRRS virus (PRRSV) has been divided into two species, including the Be-taarterivirus suid 1 (formerly PRRSV-1 or genotype 1, Type 1 or European—EU) and Be-taarterivirus suid 2 (formerly PRRSV-2, or genotype 2, Type 2 or North American—NA) types, which roughly share 60% nucleotide identity [3]. The phylogenetic tree analyses showed that the HP-PRRSV isolated from Vietnam are closed relationship to the HP-PRRSV strains circulated in China. However, variations in virulence exist, as the Vietnamese strains exhibit differing levels of virulence compared to their Chinese counterparts [7,22,23]. The re-emergence of HP-PRRSV strains with diverse virulence has been observed multiple times, both within PRRSV-1 and PRRSV-2, as well as in atypical PRRSV strains from China [22,23,24,25]. This suggests that the HP-PRRSV continues to evolve through repeated recombination and the accumulation of mutations [24,25]. Therefore, it is crucial to understand the pathogenesis of the disease caused by the HP-PRRSV strain. In our study, we investigated the pathogenicity of the PRRSV that emerged in Vietnam in 2024, using experimentally infected piglets, and assessed the innate immune response in pigs infected with HP-PRRSV in Vietnam.

The viremia and viral shedding exhibited high viral titers in whole blood and nasal swabs between 6 and 12 dpi, and the HP-PRRSV genome remained detectable up to 21 dpi. Previous studies have indicated that the duration of viremia varies depending on the virulence of PRRSV strains and the age of the host [26]. The HP-PRRSV genome can be detected as early as 24 h post-infection (hpi) and may persist for up to one month, with peak viremia typically occurring between 7 and 14 dpi [18,23,27]. The clearance of the virus from the bloodstream has been associated with the activation of cell-mediated immunity and the development of neutralizing antibodies during the second to third week post-infection [26,28,29]. In the present study, PRRSV-specific and virus-neutralizing antibodies were detectable in serum by 6 dpi, suggesting active viral replication and the initiation of an early humoral immune response. Consistent with previous reports, robust antibody responses as early as 3–7 dpi have been observed by other investigators [18,23,30]. Furthermore, antibody responses observed between 7 and 21 dpi were predominantly non-neutralizing, whereas neutralizing antibodies appeared later, even after the resolution of viremia [31]. These findings suggest that clinical samples for PRRSV diagnosis should ideally be collected within the first two weeks post-infection, as later samples may be unsuitable for viral detection by RT-PCR or virus isolation.

The clinical signs observed in this study were similar to those previously described in piglets inoculated with HP-PRRSV in China and other countries [22,23,25,29]. However, the severity of clinical signs varied among individual piglets. Gross lesions were primarily found in the lungs and lymphoid organs of piglets infected with the HP-PRRSV NA01/2024 isolate. Similarly, recent studies on the pathology of HP-PRRS infection in piglets using Indian and Chinese virus strains have shown that pathological lesions first appear in the lungs, lymph nodes, and spleen [26,31,32]. The observation of hemorrhage and infarction in the spleen in this study is consistent with previous reports involving piglets infected with HP-PRRSV [18,23,25,27,33]. Our findings indicate that piglets infected with the HP-PRRSV NA01/2024 field isolate from Vietnam exhibited characteristic lesions mainly in the lungs and lymphoid organs, along with specific pathological features typical of HP-PRRSV infection.

White blood cells (WBCs), red blood cells (RBCs), hemoglobin levels, lymphocytes, and platelets play essential roles in the immune response to control and eliminate pathogen infections [34,35,36,37]. The WBC count is a strong indicator of infectious and inflammatory diseases such as FMDV, CSFV, porcine epidemic diarrhea virus (PEDV), transmissible gastroenteritis virus (TGEV), *Haemophilus parasuis*, or PRRSV. RBCs are also involved in the immune response to pathogens. Their ability to bind and capture antigens and pathogens, along with their immunoregulatory functions, makes them crucial participants in the host’s overall defense against infection [35,36]. Lymphocytes, on the other hand, are white blood cells that appear morphologically uniform but perform diverse immune functions. They include T cells, B cells, and natural killer (NK) cells, which play key roles in antibody production, direct cell-mediated destruction of virus-infected or tumor cells, and regulation of immune responses [34,35,36,37]. Recent research in China indicated that piglets infected with HP-PRRSV or moderately virulent NACD30-like PRRSV isolates from outbreaks in 2023 showed decreased levels of RBCs, hemoglobin, and reticulocytes, along with significantly increased monocyte counts compared to uninfected controls [23]. Consistent with these findings, our results demonstrated that pigs infected with the HP-PRRSV strain NA01/2024 in Vietnam exhibited significantly lower levels of RBCs and hemoglobin compared with uninfected control pigs, suggesting potential suppression of hematopoietic function in infected pigs. These results imply that PRRSV may influence red blood cell precursors, possibly as a direct effect of viral replication. This may contribute to clinical signs related to the respiratory system in pigs infected with HP-PRRSV, such as labored breathing and dyspnea.

On the other hand, the number of platelets was dramatically reduced in pigs infected with the HP-PRRSV isolate NA01/2024 compared with the control group after three dpi. This indicates that infection with the HP-PRRSV NA01/2024 isolate induced thrombocytopenia, which may be associated with abnormal coagulation function [23]. Moreover, infection of piglets with the HP-PRRSV NA01/2024 strain in Vietnam resulted in significant increases in lymphocyte, neutrophil, eosinophil, and monocyte levels. This immune response may help control viremia; however, it could also exacerbate tissue damage during infection [27,31,38,39]. Our results indicated lower levels of RBCs, hemoglobin, and reticulocytes, along with a notable increase in lymphocytes and monocytes, compared with the control groups. These hematological alterations may contribute to the clinical signs and pathological lesions observed in pigs inoculated with HP-PRRSV.

Recent molecular surveillance has revealed that PRRSV continues to evolve rapidly, with frequent recombination events and the emergence of novel lineages across Asia and other regions. In Vietnam, PRRSV-2 remains the dominant genotype, and recent outbreaks have been associated with sub-lineages closely related to lineage 8 (HP-PRRSV) and NADC30-like or NADC34-like variants, reflecting ongoing viral diversification under vaccine and immune pressures [10,11,12]. Similar evolutionary trends have been reported in China, Korea, and Thailand, indicating regional movement and genetic exchange among strains. Studies have shown that highly pathogenic strains possess unique mutations in nonstructural proteins (NSPs) that contribute to enhanced replication and suppression of host innate immune responses, particularly through inhibition of type I interferon signaling and modulation of macrophage cytokine production [16,20,24].

Cytokine production by immune cells plays a crucial role in defending against various pathogens and in mediating pathological responses [31]. The expression of cytokines is often associated with numerous diseases. Recent studies have shown that pigs infected with HP-PRRSV typically exhibit severe inflammatory clinical signs, rather than mild to moderate symptoms. Low cytokine production at both the mRNA and protein levels is associated with classical PRRSV strains that cause milder disease, whereas HP-PRRSV infection induces severe symptoms such as high fever and pneumonia, correlating with elevated cytokine levels [31]. Cytokines and chemokines—including proinflammatory, anti-inflammatory, and growth factors such as IFN-α, IL-17A, IL-10, TNF-α, IL-1β, IL-6, TGF-β, IL-8, IFN-γ, IL-12p40, IFN-β, and CCL20—play critical roles in the immune response and defense against PRRSV infection [17,23,25,27,33]. IL-1β, IL-6, IL-12p40, IL-8, IL-17A, IFN-α, and IFN-β are proinflammatory cytokines produced primarily by macrophages and monocytes that regulate immune responses and activate other immune cells. The relatively high expression levels of IL-1β, IL-6, IL-12p40, IL-8, IL-17A, IFN-α, and IFN-β mRNA detected in pigs infected with the HP-PRRSV isolate NA01/2024 between 3 and 15 dpi may contribute to the persistent fever and acute lung injury observed in infected pigs. Other studies using HP-PRRSV and moderately virulent NADC30-like PRRSV strains from China have demonstrated increased expression of TNF-α, IL-1β, IL-10, and IFN-γ mRNA in both tissues and serum, indicating that these cytokines play important roles in the immune response to PRRSV infection [23]. TNF-α, produced mainly by macrophages and T cells, is a key proinflammatory cytokine, and in our study, its expression was significantly upregulated in pigs infected with the HP-PRRSV isolate NA01/2024 at 3 dpi. IFN-γ plays a crucial role in regulating immune cell activity and enhancing antiviral defense by inhibiting viral replication and transmission. Several studies have shown that IFN-γ contributes significantly to host defense against both HP-PRRSV and moderately virulent PRRSV strains, with elevated IFN-γ levels in serum or tissues correlating with enhanced immune responses to PRRSV [23,24,40]. In our study, piglets infected with the HP-PRRSV isolate NA01/2024 exhibited significantly higher IFN-γ levels from 3 to 21 dpi compared with the control group. Conversely, IL-10 is generally regarded as an anti-inflammatory cytokine that suppresses excessive immune activation [41]. The interplay between IL-10 and IFN-γ levels plays an important role in modulating the immune response to PRRSV in pigs [26,41,42,43,44]. It appears that the immune system of pigs infected with the HP-PRRSV isolate NA01/2024 attempts to balance the inflammatory response and restore homeostasis. Moreover, TGF-β is a multifunctional cytokine that regulates proliferation, differentiation, and diverse functions in several immune cell types, including macrophages, T cells, B cells, NK cells, and Treg cells. For example, TGF-β1 plays a key role in inducing both regulatory T cells and Th17 cells from CD4^+^ T cells. Regulatory T cells suppress immune responses, whereas Th17 cells secrete proinflammatory cytokines. TGF-β also regulates apoptosis of immature or resting B cells by inhibiting NF-κB activation, thereby influencing the production of cytokines such as IL-1 family members, TNF-α, and β-defensins [45,46]. Our results indicated that upregulation of TGF-β in both whole blood and tissues may be associated with cell-to-cell activation and regulation of cytokine production. The chemokine CCL20 plays an important role in the chemotaxis of dendritic cells (DCs), effector/memory T cells, and B cells. It is critical under both homeostatic and inflammatory conditions at mucosal and epithelial surfaces and in various pathologies. CCL20 functions as a chemotactic factor that attracts lymphocytes and, to a lesser extent, neutrophils, but not monocytes. It contributes to the recruitment of both proinflammatory IL-17–producing Th17 cells and regulatory T cells (Treg) to sites of inflammation [14,47]. Pigs infected with the HP-PRRSV isolate NA01/2024 in Vietnam showed upregulation of CCL20 in both whole blood and tissues, suggesting a potential role in the regulation and interaction of Th17, Treg, and B cells in response to infection. Overall, immune responses in pigs infected with this isolate exhibited common patterns characterized by the elevation and subsequent recovery of cytokines and chemokines such as IFN-α, IFN-β, IFN-γ, TNF-α, TGF-β, IL-1β, IL-6, IL-8, IL-10, IL-12p40, IL-17A, and CCL20 mRNA. However, these responses varied in both timing and magnitude, reflecting differences in immune regulation following PRRSV infection. Our findings indicate that the increase in cytokine and chemokine expression in pigs infected with the HP-PRRSV isolate NA01/2024 was significantly higher than in the control group and may be associated with immune cell–mediated regulation in response to HP-PRRSV infection.

Our study characterized the clinical, virological, and immunological responses of piglets infected with the newly isolated Vietnamese PRRSV strain NA01/2024, which was genetically identified as belonging to the HP-PRRSV 2 lineage currently prevalent in Vietnam. Although a direct comparison with other domestic isolates was not performed, the observed clinical signs, viremia, and cytokine responses agree with earlier reports describing variable virulence among Vietnamese PRRSV field strains. Previous studies have demonstrated that local PRRSV isolates exhibit diverse pathogenic profiles, ranging from moderate to highly virulent, which appear to be influenced by nsp2 deletion patterns, lineage evolution, and potential recombination with vaccine-derived viruses [48,49]. The clinical and immunological profiles observed in NA01/2024-infected piglets reflect characteristics typical of moderately to highly pathogenic PRRSV strains, suggesting ongoing viral adaptation and genetic diversification within Vietnamese herds.

Our findings on the NA01/2024 strain are consistent with these mechanisms, showing early onset of viremia, high cytokine expression, and severe clinical outcomes typical of moderately to highly virulent PRRSV strains infection. Compared to earlier Vietnamese isolates, NA01/2024 exhibited a similar pathogenic profile but possibly stronger inflammatory activation, suggesting continued adaptation of local viruses. Recent reports have emphasized that co-infections with PCV2 or bacterial pathogens can further exacerbate PRRSV-induced immune dysregulation, a factor that should be explored in future experiments [49]. However, our findings apply only to this specific isolate and age group and should not be generalized to all herds or field conditions. The results highlight the importance of maintaining strict biosecurity to prevent virus introduction and spread within farms. They also emphasize the need for updated vaccination programs that match circulating strains and for ongoing molecular surveillance to detect emerging PRRSV variants early and guide herd health management.

Moreover, the results of the NA01/2024 PRRS virus strain in this study add to growing evidence that PRRSV evolution is shaped by regional transmission, recombination, and immune selection pressure. Continuous genomic monitoring, coupled with functional and immunological studies, is essential to understand emerging strain diversity and disease mechanisms. Thus, the pathogenic and immunological characterization of NA01/2024 provides important reference data for both vaccine improvement and regional disease control strategies against PRRSV. These findings highlight the importance of continued comparative investigations into the pathogenicity of PRRSV isolates from different geographical regions and production systems in Vietnam. Such studies are essential for elucidating the molecular and immunological determinants of virulence and for refining strategies in disease prevention, including biosecurity, vaccination, and surveillance. Future work will focus on direct pathogenicity comparisons among representative Vietnamese PRRSV isolates, integrating clinical, pathological, and immunological data to provide a more comprehensive understanding of PRRSV evolution and its implications for herd health management in Vietnam.

In summary, piglet infected with PRRSV NA01/2024 strain show that the clinical scores began to rise at 6 days post infection (dpi), peaked between 9 and 12 dpi, and gradually declined thereafter, indicating the most severe disease occurred during the early acute phase. Rectal temperature followed a similar pattern, with pigs showing fever (≥40.5 °C) from 5–12 dpi, confirming the onset of systemic infection. Viremia was first detected at 6 dpi, reached its maximum at 12 dpi, and then steadily decreased, showing effective viral clearance by the later phase. The immune response developed in parallel: serum ELISA IgG levels and neutralizing antibody titers began increasing after 9 dpi and reached their highest levels between 18–21 dpi, when viremia had already subsided. This suggests that antibody production played a key role in viral control. Cytokine mRNA expression revealed a strong activation of both pro-inflammatory (IL-6, TNF-α) and antiviral (IFN-γ, IFN-α, IFN-β) genes, peaking at 9–12 dpi, consistent with the acute immune reaction. Anti-inflammatory cytokines such as IL-10 also increased during this period, possibly to limit excessive inflammation. Overall, the results demonstrate a typical PRRSV infection pattern, characterized by early fever and viremia, followed by a robust immune response leading to recovery

## 5. Conclusions

Our results first describe the experimental infection of piglets with the HP-PRRSV NA01/2024 isolate in Vietnam in 2024, providing a detailed analysis of its pathogenesis and confirming its highly pathogenic nature. On the other hand, our results showed the changes in blood cell components and cellular and innate immune in whole blood and tissues of pigs infected with the HP-PRRSV NA01/2024 isolate.

## 6. Study Limitations

This study has several limitations that should be acknowledged. First, the sample size was relatively small, as the experiment was designed under controlled biosafety and animal welfare restrictions, which may limit the statistical power to detect smaller effects. Second, only one PRRSV strain (NA01/2024) was tested, so the findings cannot be generalized to all circulating strains or lineages in Vietnam. Third, the experiment used 6-week-old PRRSV-naïve piglets, which may respond differently from older or previously exposed pigs commonly found in field herds. Fourth, the trial was performed under controlled laboratory conditions, which do not fully reflect the complex environmental and management factors influencing disease outcomes on farms. Possible confounders include undetected subclinical infections with other pathogens, such as PCV2 or Mycoplasma hyopneumoniae, even though all pigs tested negative before the trial. Environmental stressors, such as minor variations in temperature, humidity, or handling stress, could also have influenced immune responses and clinical scores. Furthermore, because no co-infection models were used, potential interactions between PRRSV and other endemic pathogens were not assessed. These factors may slightly affect the magnitude or variability of the observed immune and clinical responses. Despite these limitations, this study provides valuable baseline data for understanding the pathogenic and immunological features of the emerging HP-PRRSV strain in Vietnam.

## Figures and Tables

**Table 1:** HP-PRRS clinical sign scores were calculated based on clinical parameters defined in the protocol published by Li et al. [18].

Category of Clinical Sign	Symptom	Score
Systemic:	Normal	0
	Apathy	1
	Anorexia	2
	Severe depression	5
	Death	10
Respiratory:	Normal	0
	Sneezing, Coughing	1
	Tachypnea	2
	Labored breathing	3
	Abdominal breathing	5
Other symptoms:	Normal	0
	Diarrhea	1
	Conjunctivitis	2
	Reddening of the ears	3
	Skin cyanopathy	5

**Table 2:** Sequences of oligonucleotide primers for qRT-PCR analysis.

Genes	Oligonucleotide Sequences (5′-3′)	GenBank Acc. No
IL-1β	F: TGAAGAGAGAAGTGGTGTTCTGC	AJ747049
	R: GGTACAGATTCTTTCCCTTGATCC	
IL-6	F: GCATCCACTTCCAGGCCA	AF518322.1
	R: CTTCCTCATCTTCATCGTCA	
IL-8	F: TGCACTTACTCTTGCCAGAACTG	NM_213867.1
	R: CAAACTGGCTGTTGCCTTCTT	
IL-10	F: GCCTTCGGCCCAGTGAA	NM_214041.1
	R: AGAGACCCGGTCAGCAACAA	
IL-12p40	F: GATGCTGGCCAGTACACC	U08317
	R: TCCAGCACGACCTCAATG	
IL-17A	F: CAAGCGGTGGCGTTTTGCCT	NM_001005729.1
	R: GTCTCCGTCGGGGATGGGCT	
IFN-α	F: CCCCTGTGCCTGGGAGAT	XM_003480507.1
	R: AGGTTTCTGGAGGAAGAGAAGGA	
IFN-β	F: AGTTGCCTGGGACTCCTCAA	GQ415073.1
	R: CCTCAGGGACCTCAAAGTTCAT	
IFN-γ	F: CAGCTTTGCGTGACTTTGTG	X53085
	R: GATGAGTTCACTGATGGCTTT	
TNF-α	F: CGTTGTAGCCAATGTCAAAGCC	X54859
	R: TGCCCAGATTCAGCAAAGTCCA	
TGF-β	F: GAACAAACTCTTGGGCAATG	NM_214015.2
	R: ACTTCCAGCTTGTCACCTTG	
CCL-20	F: TGCTCCTGGCTGCTTTGATGTC	NM_001024589
	R: TCATTGGCGAGCTGCTGTGTG	
β-Actin	F: CTGGCATTGTCATGGACTCT	U07786
	R: GCGATGATCTTGATCTTCAT	
PRRS Realtime RT-PCR	F: ATG ATG RGC TGG CAT TCT	WOAH/TCVN
	R: ACA CGG TCG CCC TAA TTG	
	FAM-TGT GGT GAA TGG CAC TGA TTG ACA-BHQ1	

**Table 3:** Mean (sd) hematological parameters values of pigs infected with HP-PRRSV NA01/2024 strain and control groups.

Items		Reference			Day Post-Infection (dpi)							
		Rank	Unit		D0	D3	D6	D9	D12	D15	D18	D21
White Blood cells	WBC	10.20–30.00	10^9^/L	Control	18.17 ± 3.73	17.45 ± 1.61	20.97 ± 3.39	21.38 ± 1.95	18.16 ± 2.43	18.75 ± 1.80	18.35 ± 1.75	19.10 ± 2.68
				PRRS	17.63 ± 2.57	14.58 ± 2.18	10.99 ± 2.79	15.84 ± 2.55	22.94 ± 2.84	28.71 ± 3.24	29.71 ± 0.27	30.71 ± 2.75
Red blood cells	RBC	5.50–9.00	10^12^/L	Control	7.04 ± 0.74	7.19 ± 0.38	6.93 ± 0.48	7.11 ± 0.53	7.02 ± 0.53	7.21 ± 0.36	7.20 ± 0.53	7.22 ± 0.65
				PRRS	7.01 ± 0.69	6.51 ± 0.47	6.54 ± 0.58	5.56 ± 0.38	6.60 ± 0.39	7.04 ± 0.44	7.28 ± 0.33	7.04 ± 0.65
Hemoglobin	HGB	100–160	g/L	Control	120.20 ±8.23	122.00 ± 5.31	124.25 ± 6.63	121.50 ± 7.63	120.00 ± 6.29	120.60 ± 6.50	121.20 ± 8.25	121.00 ± 7.67
				PRRS	123.86 ± 9.74	121.49 ± 7.65	105.20 ± 6.02	96 ± 1.85	101.00 ± 2.06	110 ± 1.70	118 ± 2.00	115.00 ± 7.65
Hematocrit	HCT	0.330–0.520		Control	0.36 ± 0.03	0.36 ± 0.02	0.38 ± 0.02	0.38 ± 0.02	0.40 ± 0.01	0.39 ± 0.00	0.39 ± 0.00	0.38 ± 0.00
				PRRS	0.38 ± 0.00	0.32 ± 0.02	0.30 ± 0.03	0.283 ± 0.02	0.31 ± 0.02	0.31 ± 0.02	0.319 ± 0.02	0.31 ± 0.02
Mean Corpuscular Volume	MCV	51.0–73.0	fL	Control	52.60 ± 3.13	53.88 ± 2.27	53.98 ± 3.42	52.83 ± 2.10	54.98 ± 1.81	54.74 ± 2.44	54.10 ± 2.28	52.96 ± 2.45
				PRRS	53.23 ± 2.25	52.34 ± 3.59	51.11 ± 3.48	51.22 ± 2.95	52.43 ± 3.27	53.25 ± 3.76	53.56 ± 2.90	52.70 ± 3.13
Mean Corpuscular Hemoglobin	MCH	14.0–22.0	pg	Control	16.32 ± 0.77	16.62 ± 0.86	17.13 ± 0.78	16.75 ± 0.35	16.20 ± 0.33	16.18 ± 0.52	16.08 ± 0.45	16.76 ± 0.71
				PRRS	16.19 ± 0.65	16.06 ± 0.81	16.06 ± 0.53	16.4 ± 0.59	15.60 ± 0.48	16.4 ± 0.26	15.5 ± 0.25	16.40 ± 0.66
Mean Corpuscular Hemoglobin concentration	MCHC	300–360	g/L	Control	322.12 ±12.36	323.02 ± 15.48	331.28 ± 19.03	332.48 ± 10.32	323.84 ± 9.23	318.72 ± 8.90	321.88 ± 4.71	316.80 ± 2.86
				PRRS	317.77 ± 10.13	318.54 ± 12.56	326.66 ± 12.37	312 ± 16.01	282.00 ± 17.57	312 ± 16.69	285 ± 19.50	312.00 ± 13.37
Platelet	PLT	200–1000	10^9^/L	Control	245.00 ±36.09	236.40 ± 32.67	288.75 ± 22.21	306.25 ± 78.60	271.00 ± 76.15	259.40 ± 69.28	269.40 ± 56.60	253.80 ± 42.44
				PRRS	253.81 ± 44.39	218.60 ± 33.88	203.40 ± 61.01	198 ± 36.41	213.00 ± 40.55	229 ± 26.40	234 ± 12.00	229.00 ± 47.38
Neutrophil	Neu	2.80–16.10	10^9^/L	Control	5.18 ± 0.67	5.15 ± 1.89	4.96 ± 1.47	4.93 ± 2.00	4.86 ± 1.10	4.80 ± 0.67	4.28 ± 0.80	5.34 ± 0.77
				PRRS	6.04 ± 0.69	9.23 ± 1.87	9.56 ± 0.72	8.34 ± 1.79	6.35 ± 1.31	5.87 ± 1.27	5.28 ± 1.34	5.23 ± 1.64
Lymphocyte	Lym	4.80–16.20	10^9^/L	Control	11.92 ± 2.09	12.10 ± 2.22	12.09 ± 1.66	12.15 ± 1.69	10.86 ± 1.67	11.29 ± 1.92	11.94 ± 2.37	11.84 ± 1.84
				PRRS	12.26 ± 1.67	10.17 ± 1.78	9.78 ± 1.92	8.65 ± 1.71	12.04 ± 1.83	13.46 ± 2.11	14.1 ± 2.28	14.46 ± 1.86
Monocytes	Mon	0.20–2.25	10^9^/L	Control	1.44 ± 0.42	1.30 ± 0.25	1.46 ± 0.42	1.33 ± 0.36	1.36 ± 0.19	1.50 ± 0.19	1.34 ± 0.07	1.48 ± 0.07
				PRRS	1.62 ± 0.42	1.92 ± 0.44	2.01 ± 0.41	1.74 ± 0.20	1.79 ± 0.22	1.7 ± 0.09	1.86 ± 0.11	1.70 ± 0.43
Eosinophil	Eos	0.00–1.80	10^9^/L	Control	0.19 ± 0.10	0.20 ± 0.11	0.22 ± 0.17	0.22 ± 0.10	0.24 ± 0.12	0.22 ± 0.12	0.21 ± 0.12	0.20 ± 0.15
				PRRS	0.26 ± 0.13	0.78 ± 0.10	0.84 ± 0.16	1.13 ± 0.10	1.22 ± 0.08	1.12 ± 0.09	1.19 ± 0.00	1.06 ± 0.14
Basophil	Bas	0.00–0.46	10^9^/L	Control	0.29 ± 0.07	0.31 ± 0.04	0.29 ± 0.06	0.27 ± 0.07	0.29 ± 0.06	0.26 ± 0.05	0.26 ± 0.04	0.26 ± 0.04
				PRRS	0.31 ± 0.04	0.35 ± 0.05	0.39 ± 0.06	0.33 ± 0.04	0.30 ± 0.05	0.298 ± 0.05	0.29 ± 0.01	0.31 ± 0.05

## Data Availability

The original contributions presented in the study are included in the article. Further inquiries can be directed to the corresponding authors.

## Abbreviations

The following abbreviations are used in this manuscript:

PRRSV	Porcine reproductive and respiratory syndrome virus
HP-PRRSV	Highly pathogenic PRRS
LP	Low-pathogenic PRRSV
PAMs	Porcine alveolar macrophages
PBMCs	Peripheral blood mononuclear cells
RT-qPCR	Quantitative real-time reverse transcription PCR
ELISA	Enzyme-linked immunosorbent assay
RPMI	Roswell Park Memorial Institute
SVN	Serum virus-neutralizing
TNF-α	Tumor necrosis factor-alpha
mAbs	Monoclonal antibodies
RBCs	Red blood cells
TCID	Tissue culture infective dose
IFN	Interferon
IL	Interleukin
MHC	Major histocompatibility complex
CPE	Cytopathic effect
DMEM	Dulbecco’s modified Eagle’s medium
Dpc	Days post-challenge
FBS	Fetal bovine serum
ANOVA	Analysis of variance
SEM	Standard error of the mean
CCL	Chemokine (C-C motif) ligand
CXCR	C-X-C chemokine receptor
